# Glatiramer acetate does not protect from acute ischemic stroke in mice

**DOI:** 10.1186/2040-7378-6-4

**Published:** 2014-02-27

**Authors:** Peter Kraft, Kerstin Göbel, Sven G Meuth, Christoph Kleinschnitz

**Affiliations:** 1 Department of Neurology, University Hospital Würzburg, Josef-Schneider-Str. 11, 97080 Würzburg, Germany; 2 Institute for Clinical Epidemiology and Biometry and Comprehensive Heart Failure Centre, University of Würzburg, Würzburg, Germany; 3 Department of Neurology, University of Münster, Albert-Schweitzer-Campus 1, Gebäude A1, Westturm, Ebene 05 48149 Münster, Germany; 4 Institute of Physiology – Neuropathophysiology, University of Münster, Münster, Germany

**Keywords:** Glatiramer acetate, Stroke, Inflammation, Neurodegeneration

## Abstract

**Background:**

The role of the immune system in the pathophysiology of acute ischemic stroke is increasingly recognized. However, targeted treatment strategies to modulate immunological pathways in stroke are still lacking. Glatiramer acetate is a multifaceted immunomodulator approved for the treatment of relapsing-remitting multiple sclerosis. Experimental studies suggest that glatiramer acetate might also work in other neuroinflammatory or neurodegenerative diseases apart from multiple sclerosis.

**Findings:**

We evaluated the efficacy of glatiramer acetate in a mouse model of brain ischemia/reperfusion injury. 60 min of transient middle cerebral artery occlusion was induced in male C57Bl/6 mice. Pretreatment with glatiramer acetate (3.5 mg/kg bodyweight) 30 min before the induction of stroke did not reduce lesion volumes or improve functional outcome on day 1.

**Conclusions:**

Glatiramer acetate failed to protect from acute ischemic stroke in our hands. Further studies are needed to assess the true therapeutic potential of glatiramer acetate and related immunomodulators in brain ischemia.

## Findings

For many years ischemic stroke has been regarded as a mere thrombo-embolic disease. However, there is increasing evidence that the immune system is also critically involved in stroke occurrence and development [1,2]. Brain ischemia triggers rapid activation of cerebral endothelial cells and the release of danger signals from dying neuronal tissues. As a consequence, different cell adhesion receptors are upregulated and soluble chemoattractants such as chemokines and cytokines are secreted that guide the targeted invasion of innate immune cells (neutrophils, macrophages) to the sites of tissue damage [2]. These peripheral immune cells in concert with resident cell populations like microglia or astrocytes produce a potpourri of potentially harmful mediators (e.g. reactive oxygen species, degrading enzymes) and this sterile inflammation further perpetuates the ischemic cascade. Interestingly, T lymphocytes, which belong to the adaptive immune system, can likewise foster ischemic brain damage. *Rag1*^*−/−*^ mice lacking functional T lymphocytes develop dramatically smaller brain infarctions and less severe functional deficits after transient middle cerebral artery occlusion [3-5]. Of note, distinct T cell subsets for instance regulatory T cells (Treg) or γδ T cells exist that are particularly harmful in stroke [5]. Given that the immune system is of significant relevance for the pathophysiology of acute ischemic stroke, it seems reasonable to evaluate the safety and efficacy of specific immunomodulatory compounds in terms of stroke prevention and treatment.

Glatiramer acetate (GA) is a synthetic peptide that consists of four amino acids in a fixed molar residue ratio [6]. The substance for many years is in clinical use for the treatment of relapsing-remitting multiple sclerosis and has demonstrated to suppress experimental allergic encephalomyelitis (EAE), the most common animal model of multiple sclerosis [6]. The exact mode of action of GA in autoimmune neuroinflammation is still unclear but among other things is based on its ability to inhibit Th1 proinflammatory cytokines and to induce Th2 cell and Treg activation or to reduce monocyte reactivity [7]. Direct neuroprotective effects mediated for instance by brain-derived neurotrophic factor (BDNF) may also play a role [8]. However, the therapeutic potential of GA in ischemic stroke is unclear and previous studies in rodents produced controversial results [9,10]. Here, we investigated the effect of GA on stroke outcome in a well-established mouse model of brain ischemia/reperfusion injury.

All experiments were approved by the respective institutional (University of Würzburg, Germany; University of Münster, Germany) and governmental authorities (Regierung von Unterfranken and Landesamt für Natur, Umwelt und Verbraucherschutz Nordrhein-Westfalen). Six to eight week old male C57Bl/6 mice weighing 20–25 g were used in the study which was conducted in accordance with the recently published ARRIVE guidelines (http://www.nc3rs.org/ARRIVE). 60 min of transient middle cerebral artery occlusion (tMCAO) using a monofilament was performed as described [11]. This model mounts a strong and immediate local inflammatory response in the ischemic brain [12]. GA (TEVA Pharma, 3.5 mg/kg bodyweight) [13] or vehicle (NaCl 0.9%) were injected intravenously 30 min before tMCAO. Animals were randomly assigned to the operators by an independent person not involved in data acquisition and analysis. We performed surgery and evaluation of all read-out parameters while being blinded to the experimental groups. Infarct volumes were calculated from coronal brain slices stained with 2,3,5-triphenyltetrazolium chloride (TTC) 24 h after tMCAO [11]. The Bederson score [14] and the grip test score [15] were assessed to monitor global neurologic function, motor function, and coordination.

Stroke volumes were expressed as mean ± SEM. Functional outcome parameters were depicted as scatter plots including median with the 25% percentile and the 75% percentile given in brackets in the text. For statistical analysis, the GraphPad Prism 5.0 software package (GraphPad Software) was used. Data were tested for Gaussian distribution with the D’Agostino and Pearson omnibus normality test and then analyzed by unpaired, two-tailed Student´s t-test or Mann Whitney U test. P values < 0.05 were considered statistically significant.

**Figure 1:**
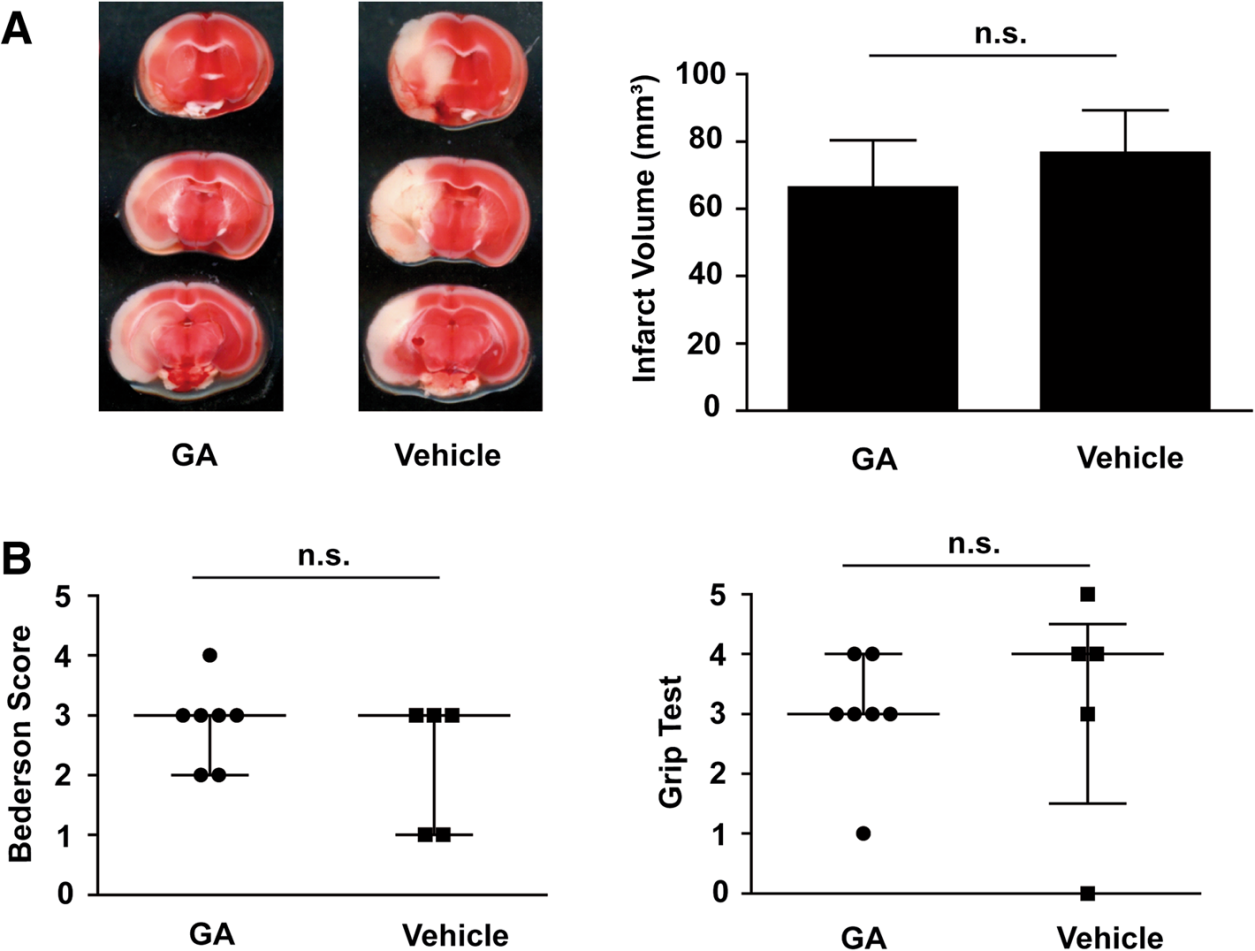
**Glatiramer acetate (GA) does not protect from acute brain ischemia/reperfusion injury. (A)** (Left panel) Representative 2,3,5-triphenyltetrazolium chloride stains of 3 corresponding coronal brain sections of a GA-treated mouse and a vehicle-treated mouse sacrificed on day 1 after transient middle cerebral artery occlusion (tMCAO). GA was applied 30 min before tMCAO at a dosage of 3.5 mg/kg bodyweight. Stroke sizes appeared to be similar between the two groups and this was confirmed by infarct volumetry (right panel) (n = 7/group). **(B)** GA did not improve functional outcome on day 1 after tMCAO. The Bederosn score (left panel) and the grip test (right panel) did not differ between GA-treated mice and vehicle-treated controls (n = 5-7/group). Unpaired, 2-tailed Student’s *t* test (infarct volumes) or Mann–Whitney Test (Bederson score, grip test); n.s. = not significant.

Prophylactic administration of GA did not alter stroke outcome (Figure 1). Stroke volumes on day 1 after 60 min tMCAO were similar between GA-treated mice and vehicle-treated controls (66.4 mm^3^ ± 37.3 mm^3^ vs. 76.7 mm^3^ ± 31.0 mm^3^, n = 7, p > 0.05) (Figure 1A). Accordingly, no differences in functional outcome parameters were observed (Bederson score: 3.0 [2.0, 3.0] vs 3.0 [1.0, 3.0], n = 5–7, p > 0.05; Grip test: 3.0 [3.0, 4.0] vs 4.0 [1.5, 4.5], n = 5–7, p > 0.05) (Figure 1B).

The present study failed to demonstrate a protective effect of GA in experimental stroke in mice even when administered in a prophylactic setting. This is in line with a previous investigation showing that GA does not reduce stroke volumes or functional deficits on day 3 and day 7 after transient or permanent MCAO despite a downregulation of pro-inflammatory cytokines [10]. In contrast, Ibarra et al. [9] reported that GA exerts beneficial effects on stroke outcome both histologically and clinically after tMCAO in rats when applied 30 min after reperfusion. However, in this study GA-induced neuroprotection became manifest not before day 7. The exact reasons for these discrepant findings are unclear at present but differences in the stroke models (transient vs permanent) or animal species (rat vs mice) used certainly play a role. In addition, the dosing regimens including the time of GA-application (prophylactic vs therapeutic) differed between the two ancestor studies and ours.

GA has already been tested in other neurodegenerative disease models apart from stroke and EAE but again with divergent results [16]. While GA protected from axonal and neuronal degeneration after optic nerve crush [17,18] or in a mouse model of Alzheimer’s disease [19], the substance was ineffective in animal models of amyotrophic lateral sclerosis [20,21]. However, even if GA might mediate some neuroprotection in primarily neurodegenerative diseases its application in ischemic stroke appears less promising.

Several studies already exist that addressed the therapeutic potential of other immunomodulatory agents in models of ischemic stroke. Treatment with FTY720, which sequesters T lymphocytes within their lymphoid organs [22] and which is also approved for the treatment of multiple sclerosis, protects from ischemic neurodegeneration by reducing the interplay between thrombotic and inflammatory processes (‘thrombo-inflammation’) in the cerebral microvasculature [11]. However, other modes of FTY720 action such as blood–brain barrier stabilization, reduction of immune cell infiltration or direct neuroprotection might also play a role [23,24]. Similarly, blocking of the very late antigen-4/vascular adhesion molecule-1 axis by injecting a monoclonal antibody against CD49d (the murine equivalent of natalizumab) has been shown to shield the brain against deleterious neuroinflammation after stroke in rodents [25,26] although an identical approach was ineffective in our hands (unpublished observations).

Our study has several limitations. First, we only focused on the outcome at a very early stage after stroke (day 1). Therefore, we cannot exclude neuroprotective effects of GA that become operative only at more advanced stages of infarct development [9]. Moreover, different GA dosing or application regimes could have produced different results. Finally, we refrained from conducting profound mechanistic studies since we did not find an obvious phenotype in GA-treated animals.

Taken together, our study failed to confirm a protective effect of GA in acute ischemic stroke in mice. Nevertheless, the concept of immunomodulation in brain ischemia is still tempting given the promising reports on other immunomodulatory agents. Further studies in relevant disease models are warranted.

## Competing interests

The authors declare that they have no competing interests.

## Authors’ contributions

PK and KG performed the experiments and drafted the manuscript. SGM and CK conceived and funded the entire study and wrote the manuscript. All authors read and approved the final manuscript.
